# Utility of qSOFA and modified SOFA in severe malaria presenting as sepsis

**DOI:** 10.1371/journal.pone.0223457

**Published:** 2019-10-09

**Authors:** Prapit Teparrukkul, Viriya Hantrakun, Mallika Imwong, Nittaya Teerawattanasook, Gumphol Wongsuvan, Nicholas PJ. Day, Arjen M. Dondorp, T. Eoin West, Direk Limmathurotsakul

**Affiliations:** 1 Medical Department, Sunpasitthiprasong Hospital, Ubon Ratchthani, Thailand; 2 Mahidol Oxford Tropical Medicine Research Unit, Faculty of Tropical Medicine, Mahidol University, Bangkok, Thailand; 3 Department of Molecular Tropical Medicine and Genetics, Faculty of Tropical Medicine, Mahidol University, Bangkok, Thailand; 4 Medical Technology Department, Sunpasitthiprasong Hospital, Ubon Ratchthani, Thailand; 5 Centre for Tropical Medicine and Global Health, University of Oxford, Oxford, United Kingdom; 6 Division of Pulmonary and Critical Care Medicine, Department of Medicine, University of Washington, Seattle, Washington, United States of America; 7 Department of Global Health, University of Washington, Seattle, Washington, United States of America; 8 Department of Tropical Hygiene, Faculty of Tropical Medicine, Mahidol University, Bangkok, Thailand; University of Pittsburgh, UNITED STATES

## Abstract

Sepsis can be caused by malaria infection, but little is known about the utility of the quick Sequential (Sepsis-Related) Organ Failure Assessment (qSOFA) and SOFA score in malaria. We conducted a prospective observational study from March 2013 to February 2017 to examine adults admitted with community-acquired infection in a tertiary-care hospital in Ubon Ratchathani, Northeast Thailand (Ubon-sepsis). Subjects were classified as having sepsis if they had a modified SOFA score ≥2 within 24 hours of admission. Serum was stored and later tested for malaria parasites using a nested PCR assay. Presence of severe malaria was defined using modified World Health Organization criteria. Of 4,989 patients enrolled, 153 patients (3%) were PCR positive for either *Plasmodium falciparum* (74 [48%]), *P*. *vivax* (69 [45%]), or both organisms (10 [7%]). Of 153 malaria patients, 80 were severe malaria patients presenting with sepsis, 70 were non-severe malaria patients presenting with sepsis, and three were non-severe malaria patients presenting without sepsis. The modified SOFA score (median 5; IQR 4–6; range 1–18) was strongly correlated with malaria severity determined by the number of World Health Organization severity criteria satisfied by the patient (Spearman’s rho = 0.61, p<0.001). Of 80 severe malaria patients, 2 (2.5%), 11 (14%), 62 (77.5%) and 5 (6%), presented with qSOFA scores of 0, 1, 2 and 3, respectively. Twenty eight-day mortality was 1.3% (2/153). In conclusion, qSOFA and SOFA can serve as markers of disease severity in adults with malarial sepsis. Patients presenting with a qSOFA score of 1 may also require careful evaluation for sepsis; including diagnosis of cause of infection, initiation of medical intervention, and consideration for referral as appropriate.

## Introduction

Sepsis is a life-threatening acute organ dysfunction caused by a dysregulated host response to infection with any organism; including bacterial, fungal, viral and parasitic agents [1, 2]. Globally, about 20 million cases of sepsis and 5.3 million deaths from sepsis are estimated to occur annually, with most of the burden in low and middle-income countries (LMICs) [3]. In 2016, the Sepsis-3 Task Force proposed that in patients with suspected infection an increase of 2 points in the Sequential (Sepsis-Related) Organ Failure Assessment (SOFA) score could serve as clinical criteria for sepsis [1]. In addition, a “quick SOFA” (qSOFA) score of 2 or greater should be used to prompt clinicians at the bedside to suspect sepsis, further investigate for organ dysfunction and cause of infection, and initiate or escalate therapy as appropriate, because those patients are more likely to have poor outcomes [1].

In malaria endemic areas, malaria patients are also at risk of developing and presenting as sepsis [4]. In 2016, the World Health Organization (WHO) reported that there were 216 million cases of malaria worldwide and 445,000 cases died [5]. However, the mechanisms that lead to life-threatening organ failure from malaria may differ from those of classic bacterial sepsis [6, 7]. Severe malaria can be identified using a definition proposed by the WHO [8], representing those who are at high risk of death due to malaria. A recent study showed that malaria patients with organ failure (defined as qSOFA ≥2) are also at high risk of death [2]. The study also found that patients presenting with a qSOFA score of 1 was associated with increased risk of death, an observation that may have important implications for triage in low-resource settings [2].

Nonetheless, it is largely unknown whether a significant proportion of severe malaria patients may present with a qSOFA score of 0 or 1. Such patients may be considered not to have sepsis, and as a result not afforded further investigation of the cause of infection or close management [1]. To further evaluate the clinical epidemiology of malaria patients presenting as sepsis and the correlation between qSOFA, SOFA and malaria severity, data from adults with malaria infection prospectively enrolled in a study evaluating community-acquired infection in northeast Thailand were analyzed.

## Materials and methods

### Study design and study site

We conducted a four-year prospective observational study from March 2013 through February 2017 (NCT02217592) to examine the epidemiology and outcomes of individuals with community-acquired infection and accompanying systemic manifestations of infection in a resource-limited setting of Sunpasitthiprasong Hospital in Ubon Ratchathani province, Northeast Thailand [9]. Thailand is an upper-middle income country, spending $264 on health per capita in 2013 [10]. Ubon Ratchathani is the second largest province in Northeast Thailand, and is bordered by Cambodia to the south and Laos to the east. Sunpasitthiprasong Hospital acts as a referral hospital to 25 district hospitals (secondary-care hospitals) in the province, Tambon Health Promoting Hospitals (primary-care hospitals) in the main district (Amphoe Muang) of the province, and hospitals in adjacent provinces [9].

### Study participants

From March 2013 to February 2017, we prospectively enrolled adult patients aged 18 years and older who were admitted with a primary diagnosis of suspected or documented infection made by the attending physician (Ubon-sepsis) [9]. For inclusion, enrollment had to occur within 24 hours of admission to the study hospital, and patients required the presence of at least three systemic manifestations of infection documented in the medical record. The 20 systemic manifestations were consolidated from the 22 variables proposed as diagnostic criteria for sepsis by Surviving Sepsis Campaign (SSC) 2012 (S1 Table) [11]. We excluded patients who were suspected of having hospital-acquired infections determined by the attending physician, were hospitalized within 30 days prior to the current admission, or were transferred from other hospitals with a total duration of hospitalization >72 hours. Patients enrolled in the Ubon-sepsis study from March 2013 to February 2014 were part of the priori analysis presented by Rudd et al [2].

### Study protocol

The study team of trained research nurses sequentially screened all medical patients by conducting ward rounds and reviewing admission logs in the emergency department, medical wards and medical ICUs twice daily (morning and afternoon) on each working day. Nurses in the emergency department also notified the study team directly about potentially eligible patients. Written, informed consent was obtained from participants prior to enrollment. For illiterate participants, the study information was read to the participant and their impartial witness, then fingerprinted and signed informed consent was obtained from the participant and their representative, respectively, before enrollment.

Following enrollment, patients were evaluated by the study nurses at the bedside using four point-of-care assessments: a whole blood lactate Rapid Diagnostic test (RDT) (Lactate Pro 2, Arkray Global Business Inc., Australia), a whole blood glucose RDT (ACCU-CHECK Performa, Roche Diagnostic, Germany), pulse oximetry (Nellcor N-65, Covidien plc., Ireland) and the Glasgow Coma Scale (GCS). The results were reported to the attending physicians. The study did not involve any clinical interventions and all medical treatment was provided by the attending physicians and respective medical teams. Twenty eight-day mortality data were collected via telephone contact if subjects were no longer hospitalized and had been discharged alive.

### PCR assays for malaria diagnosis

The FilmArray Instrument designed by BioFire Diagnostic (Salt Lake City, UT) and the Severe Acute Systemic Febrile Illness (SASFI) pouch containing various species of *Plasmodium* were used as described previously [12]. In short, the SASFI pouch used a 200 μL fresh whole blood sample in a two-stage nested PCR assay, and the sample-to-answer time was about 80 min [12]. The FilmArray result was not reported to the attending physicians as diagnostic sensitivity and specificity of FilmArray in this setting has never been evaluated. At Sunpasitthiprasong Hospital, physicians normally diagnose malaria based on blood film routinely performed with complete blood count. In this study, frozen EDTA blood samples collected on enrollment from patients with positive FilmArray malaria PCR assay results or from patients with a final diagnosis of malaria made by attending physicians were subsequently tested for malaria using conventional nested PCR assay [13]. Then, we evaluated clinical manifestations and outcomes of malaria patients confirmed with nested PCR assay.

### Ethics

We conducted the study in full compliance with the principles of good clinical practice (GCP), and the ethical principles of the Declaration of Helsinki. The study protocol and related documents were approved by Sunpasitthiprasong Hospital Ethics Committee (039/2556), the Ethics Committee of the Faculty of Tropical Medicine, Mahidol University (MUTM2012-024-01), the University of Washington Institutional Review Board (42988) and the Oxford Tropical Research Ethics Committee at the University of Oxford (OXTREC172-12).

### Definitions

Presence or absence of severe malaria was defined using modified World Health Organization (WHO) criteria (Table 1). Malaria severity (as an ordinal variable) was defined by the total number of the modified WHO criteria that a patient satisfied [8]. Sepsis was defined as an infection with organ dysfunction in accordance with the 2016 International Consensus (Sepsis-3) guidelines for sepsis [1, 14]. A modified SOFA score ≥2 was used to define organ dysfunction and was calculated as the sum of respiratory, coagulation, liver, cardiovascular, central nervous system, and renal parameters +/-24 hours of screening [1, 14]. The study was initiated in 2012 prior to the Sepsis-3 definition [1, 14], and inotropic and vasopressor agent doses were not recorded into the case record form (CRF) as previously described [9]. For the cardiovascular component of the SOFA score, the scoring was modified such that subjects were scored a maximum of 2 (on a 4-point scale) if they received only dobutamine or dopamine, and scored a maximum of 3 if they received epinephrine or norepinephrine. For the respiratory component of the SOFA score, as PaO_2_/FiO_2_ indices were not available for the majority of subjects due to infrequency of arterial blood gas tests, the score was modified as follows: Subjects were scored a maximum of 2 (4-point scale) if they received advanced respiratory support (endotracheal tube, gas powered or electrical powered mechanical ventilation) and arterial blood gas test was not performed. For patients who required mechanical ventilation, the GCS verbal score was calculated by the following formula: (-0.3756) + GCS Motor*(0.5713) + GCS Eye*(0.4233) [15]. The maximum possible modified score was 23 (Table 1). The qSOFA score was calculated as described previously [2, 14].

**Table 1:** Comparison between modified SOFA score and malaria severity.

System	Modified SOFA score	Malaria severity
Respiration*	0 if PaO_2_/FiO_2_ ≥400, 1 if PaO_2_/FiO_2_ <400, 2 if PaO_2_/FiO_2_ <300 or mechanical ventilation without arterial blood gas test, 3 if PaO_2_/FiO_2_ <200 with mechanical ventilation, and 4 if PaO_2_/FiO_2_ <100 with mechanical ventilation	1 if oxygen saturation <92% and respiratory rate >30/min, or mechanical ventilation
Coagulation	0, 1, 2, 3 and 4 if platelets ≥150,000, <150,000, <100,000, <50,000 and <20,000 /μL, respectively	N/A
Liver	0, 1, 2, 3 and 4 if bilirubin <1.2, 1.2–1.9, 2.0–5.9, 6.0–11.9 and >12.0 mg/dL, respectively	1 if bilirubin >3 mg/dL
Cardiovascular*	0, 1, 2 and 3 if mean arterial pressure ≥70, mean arterial pressure <70, dobutamine or dopamine (any dose) and epinephrine or norepinephrine (any dose), respectively	1 if systolic blood pressure <80 mmHg, or epinephrine or norepinephrine (any dose)
Central nervous system	0, 1, 2, 3 and 4 if Glasgow Coma Scale 15, 13–14, 10–12, 6–9 and <6, respectively	1 if Glasgow Coma Score <11
Renal	0, 1, 2, 3 and 4 if creatinine <1.2, 1.2–1.9, 2.0–3.4, 3.5–4.9 and >5.0 mg/dL, respectively. 3 and 4 if urine output <500 and <200 mL/d, respectively.	1 if creatinine >3 mg/dL
Acidosis	N/A	1 if venous bicarbonate <15 mmol/L
Hyperlactataemia	N/A	1 if venous lactate >4 mmol/L
Hypoglycaemia	N/A	1 if blood or plasma glucose <40 mg/dL
Severe anaemia	N/A	1 if haematocrit <20%
Generalized convulsions	N/A	1 if generalized convulsions
Hyperparasitaemia	N/A	1 if parasitaemia >10%

* For the cardiovascular component of the SOFA score, the scoring was modified such that subjects were scored a maximum of 2 if they received only dobutamine or dopamine, and scored a maximum of 3 if they received epinephrine or norepinephrine. For the respiratory component of the SOFA score, the subjects were scored a maximum of 2 if they received advanced respiratory support (endotracheal tube, gas powered or electrical powered mechanical ventilation) and arterial blood gas test was not performed.

### Statistical analysis

Data were summarized with medians and interquartile ranges (IQR) for continuous measures, and proportions for discrete measures. IQRs are presented in terms of 25^th^ and 75^th^ percentiles. Continuous variables and proportions were compared between groups using Kruskal Wallis tests and Fisher exact tests, respectively. A Spearman rank correlation coefficient (Spearman’s rho) was used to assess correlations between malaria severity and qSOFA and modified SOFA score. The McNemar exact test was used to compare sensitivity of different diagnostic criteria. Specificity of qSOFA and modified SOFA score to detect severe malaria was not estimated as the prospective study focusing on patients with community-acquired infection and sepsis [9]. All analyses were performed with STATA 14.2 (StataCorp, College Station, TX, USA). The final database with the data dictionary are publicly available online (https://dx.doi.org/10.6084/m9.figshare.9765362).

## Results

Over the 4 year study period, 4,989 patients with community-acquired infection were enrolled and evaluated (S1 Fig). From May 2015 to August 2016, 937 enrolled patients were sequentially tested for malaria infection using FilmArray platform; 25 (3%) were positive. On the entire study cohort of 4,989 patients, we evaluated final diagnoses made by attending physicians using data of The International Classification of Disease, Tenth Revision (ICD-10) collected, and found that 24 of those 25 FilmArray positive patients and another 134 patients received final diagnoses of malaria by treating clinicians (ICD-10 codes B50-B54). Of 159 patients with malaria diagnosed by FilmArray or by treating clinicians, only one did not have stored EDTA blood samples available for nested PCR assay. Of 158 patients tested for malaria parasites using nested PCR assay, 153 were positive and included in the analysis of this study.

### Baseline characteristics

Of 153 patients with nested PCR positive results, 74 (48%) were positive for *Plasmodium falciparum* (PF), 69 (45%) had *P*. *vivax* (PV), and 10 (7%) had mixed PF and PV infection (Table 2). Overall, 6 (4%) were non-transferred patients, 142 (93%) were transferred from other hospitals in the province, and 5 (3%) were transferred from hospitals in other provinces.

**Table 2:** Baseline characteristic of 153 malaria patients.

	All (n = 153)	*P*. *falciparum* patients (n = 74)	Mixed *P*. *falciparum* and *P*. *vivax* patients (n = 10)	*P*. *vivax* patients (n = 69)	P value
Sex (% male)	137 (90%)	66 (90%)	9 (90%)	62 (90%)	>0.99
Age (years) (median [IQR, range])	39 (30–51, 19–76)	39 (30–49, 20–76)	25 (21–33, 19–51)	41 (31–53, 19–71)	0.02
Transferred from other hospitals	147 (96%)	73 (99%)	10 (100%)	64 (93%)	0.23
qSOFA score ≥2 *	114 (75%)	51 (69%)	8 (80%)	55 (80%)	0.30
Sepsis (modified SOFA score ≥2)	150 (98%)	73 (99%)	10 (100%)	67 (97%)	0.68
Modified SOFA score (median [IQR, range])	5 (4–6, 1–18)	5 (4–7, 1–18)	5 (5–6, 3–6)	4 (4–5, 1–11)	0.06
Modified WHO criteria for severe malaria **					
Jaundice (total bilirubin >3 mg/dL)	41/149 (28%)	28/72 (39%)	2/10 (20%)	11/67 (16%)	0.008
Shock (systolic blood pressure <80 mmHg, or epinephrine or norepinephrine [any dose])	35 (23%)	14 (19%)	5 (50%)	16 (23%)	0.09
Renal failure (plasma creatinine >3 mg/dL)	11/152 (7%)	8/74 (11%)	0/10 (0%)	3/68 (4%)	0.33
Severe anaemia (haematocrit <20%)	10 (7%)	6 (8%)	1 (10%)	3 (4%)	0.48
Acidosis (venous bicarbonate <15 mmol/L)	7/151 (5%)	5/72 (7%)	0/10 (0%)	2/69 (3%)	0.66
Pulmonary oedema (oxygen saturation <92% and respiratory rate >30/min, or mechanical ventilation)	6 (4%)	3 (4%)	0 (0%)	3 (4%)	>0.99
Hyperparasitaemia (>10%)	6/114 (5%)	6/61 (10%)	0/6 (0%)	0/47	0.07
General convulsions	5 (3%)	3 (4%)	0 (0%)	2 (3%)	>0.99
Hyperlactataemia (venous lactate >4 mmol/L)	3 (2%)	2 (3%)	0 (0%)	1 (1%)	>0.99
Impaired consciousness (Glasgow Coma Scale <11)	2 (1%)	2 (3%)	0 (0%)	0 (0%)	0.56
Hypoglycaemia (blood or plasma glucose <40 mg/dL)	1 (1%)	1 (1%)	0 (0%)	0 (0%)	>0.99
Severe malaria (≥1 modified WHO criteria)	80 (52%)	42 (57%)	8 (80%)	30 (43%)	0.06
28-day mortality	2 (1%)	2 (3%)	0 (0%)	0 (0%)	0.56

* qSOFA = quick Sequential (Sepsis-Related) Organ Failure Assessment. qSOFA criteria is defined as respiratory rate ≥22/min, altered mentation (Glasgow Coma Scale <15) or systolic blood pressure ≤100 mmHg.

** Denominators represent the total number of patients who had been tested for those parameters.

Of 153 malaria-confirmed patients, 114 (75%) had a qSOFA score ≥2 and 150 (98%) met criteria for sepsis, with a modified SOFA score ≥2. Of overall, 80 (52%) met criteria for severe malaria, with at least one modified WHO criterion within the first 24 hours (Table 1). All 80 severe malaria patients met criteria of sepsis. The most common criteria for severe malaria found were jaundice (total bilirubin >3 mg/dl; 28% [41/149]) and shock (systolic blood pressure <80 mmHg, or need for administration of epinephrine or norepinephrine [any dose]; 23% [35/153]). Of 113 patients with parasite count data, 6 (5%) had parasitaemia >10%. Jaundice was more common among patients with PF infection (39%) than patients with mixed infection (20%) and PV infection (16%) (p = 0.008). There was borderline evidence that prevalence of severe malaria was different by the type of malaria infection (p = 0.06). The prevalence of severe malaria was highest in patients with mixed infection (80%), followed by PF (57%) and PV (43%) infection.

### Blood film for malaria and mixed infections

Of 153 malaria-confirmed patients, 151 (99%) had blood films positive for *Plasmodium* organisms at the study hospital. Of the two patients without positive blood films at the study hospital, one patient (37-year-old male) had blood film positive only at a primary care unit prior to referral, and data of parasite count was not available and blood films were repeatedly negative at the study hospital. His blood specimen was not tested with the FilmArray. The other patient (25-year-old female) had no history of blood film positive for *Plasmodium* organisms, had a positive dengue rapid test for NS1, and was diagnosed and treated as dengue infection. She improved and survived. She had PCR positive for malaria by the FilmArray platform, which was later confirmed as PV infection by nested PCR assay. We identified another patient (39-year-old male) with blood film and nested PCR assay positive for PF, who had a final diagnosis of both severe PF infection and dengue hemorrhagic shock. He had a negative dengue rapid test for NS1 and positive dengue IgM antibody. He was treated as co-infection, improved and survived. In total, only these two patients (1%; 2/153) probably had co-infection with malaria and dengue in our study.

All patients had blood cultures performed based on the study protocol. Three patients (2%) had blood cultures positive for bacterial pathogens: one patient had *Escherichia coli* bacteremia (modified SOFA score = 5, malaria severity = 2), a second patient had *Pseudomonas spp*. bacteremia (modified SOFA score = 9, malaria severity = 2), and a third had non-typhoidal *Salmonella* bacteremia (modified SOFA score = 6, malaria severity = 0). Those three patients were also treated with parenteral antibiotics and survived. The median modified SOFA score of those three patients with bacterial co-infection was not significantly higher than those who had no bacterial co-infection (6 vs. 5, p = 0.16).

### Outcomes

Of 153 malaria-confirmed patients, 2 (1.3%) died within 28 days of admission. 81% (65/80) and 59% (43/73) of patients with severe and non-severe malaria, respectively, received parenteral artesunate during hospital admission. Among 151 survivors, the median length of hospital stay at the study hospital was 3 days (IQR 2–4, range 1–17 days). All patients’ survival outcomes on day 28 were confirmed via telephone contact.

The first fatal case (43 year-old male) presented at a secondary-care hospital in Ubon Ratchathani province with high fever and a history of generalized convulsion for two times. He had no known underlying diseases. His blood film found PF >10%. He received parenteral diazepam and artesunate, and was transferred to the emergency room of the study hospital where he developed sudden cardiac arrest but was resuscitated. He had a qSOFA score of 3, modified SOFA score of 18 and malaria severity of 8. Parenteral artesunate plus oral mefloquine and parenteral ceftriaxone were prescribed at the study hospital. He died on day 2 after hospital admission. The other fatal case (66 year-old male) presented at a secondary-care hospital in Ubon Ratchathani province with high fever, jaundice, dyspnea and hypoglycemia (blood glucose level of 30 mg/dL). He had no known underlying diseases. His complete blood count showed leukocytosis (WBC 23,570/μL with 60% granulocytes and 34% lymphocytes) and mild thrombocytopenia (platelet 120,000 mg/dL). No malaria parasitemia was mentioned in the medical record. He was treated with parenteral ceftriaxone and glucose, intubated and was transferred. On admission at the study hospital, the patient had a qSOFA score of 3, modified SOFA score of 18 and malaria severity of 9. His blood film found PF >10%. Parenteral artesunate plus oral mefloquine and parenteral ceftrixone were prescribed at the study hospital. He developed acute respiratory distress syndrome (ARDS) and acute kidney injury (AKI), and died the next day.

### Correlation between qSOFA score and malaria severity

**Fig 1:**
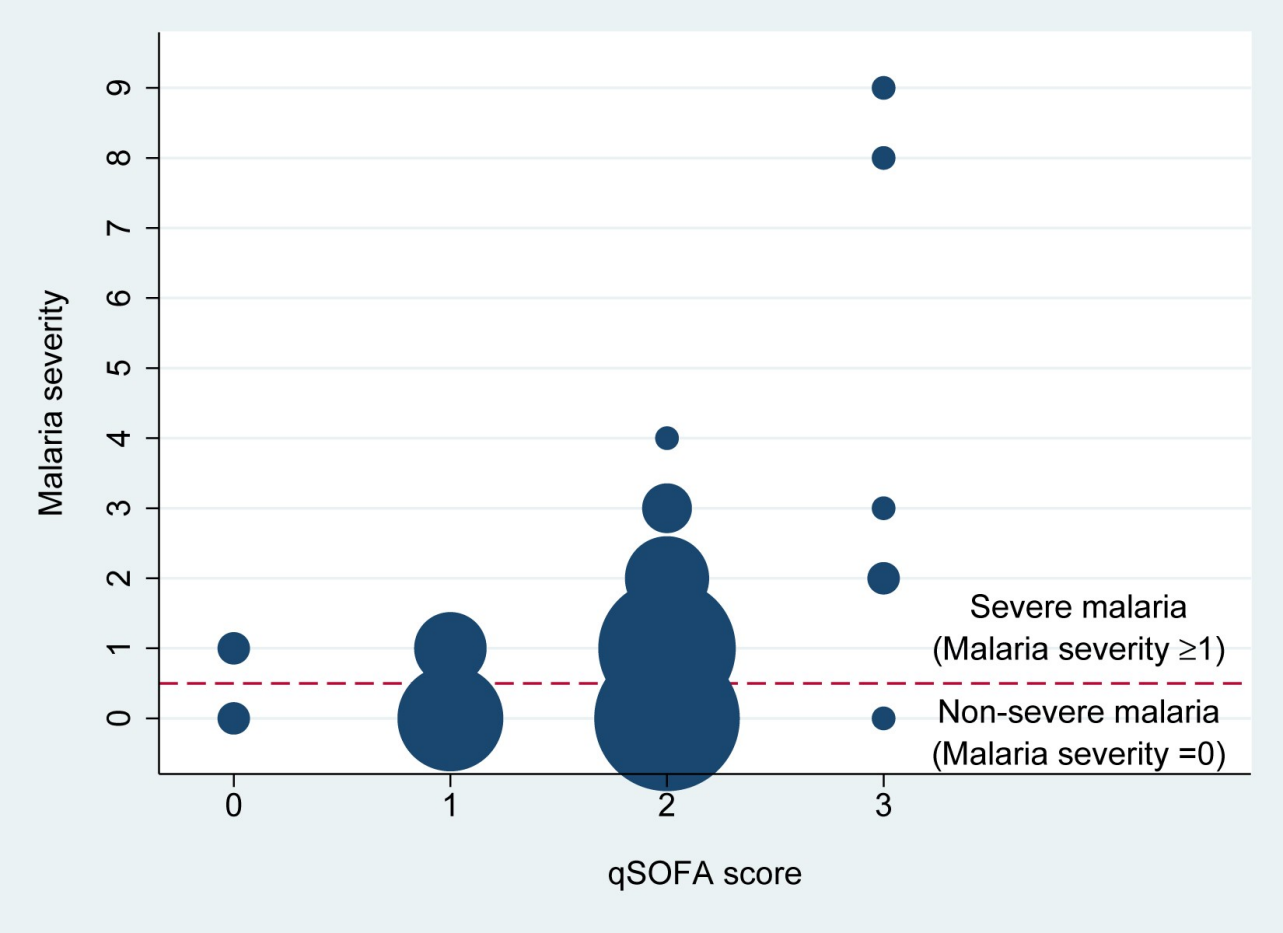
Relationship between qSOFA score and malaria severity (the total number of WHO severity criteria for severe malaria satisfied by the patient). Size of each dot represents the total number of patients with a qSOFA score and malaria severity. Each smallest dot represents one patient, and the biggest dot represents 45 patients (qSOFA score of 2 and malaria severity of 0). Both fatal patients in the study had malaria severity of ≥8.

The qSOFA score had a weak correlation with malaria severity (Spearman’s rho = 0.32, p<0.001) (Fig 1). Of 80 severe malaria patients, 2 (2.5%), 11 (14%), 62 (77.5%) and 5 (6%), presented with qSOFA scores of 0, 1, 2 and 3, respectively. The sensitivity of a qSOFA score ≥1 was significantly higher than a qSOFA score ≥2 to detect severe malaria patients (98% [95%CI 91–100%] vs. 84% [95%CI 74–91%], p = 0.001). Two patients who had qSOFA score of zero but were classified as severe malaria met the WHO criteria of jaundice. Of 73 non-severe malaria patients evaluated, 2 (3%), 24 (33%), 46 (63%) and 1 (1%) had qSOFA scores of 0, 1, 2 and 3, respectively.

A sensitivity analysis was conducted by including only the 80 severe malaria patients, and a moderate correlation between qSOFA and malaria severity was observed (Spearman’s rho = 0.45, p<0.001).

### Correlation between the modified SOFA score and malaria severity

**Fig 2:**
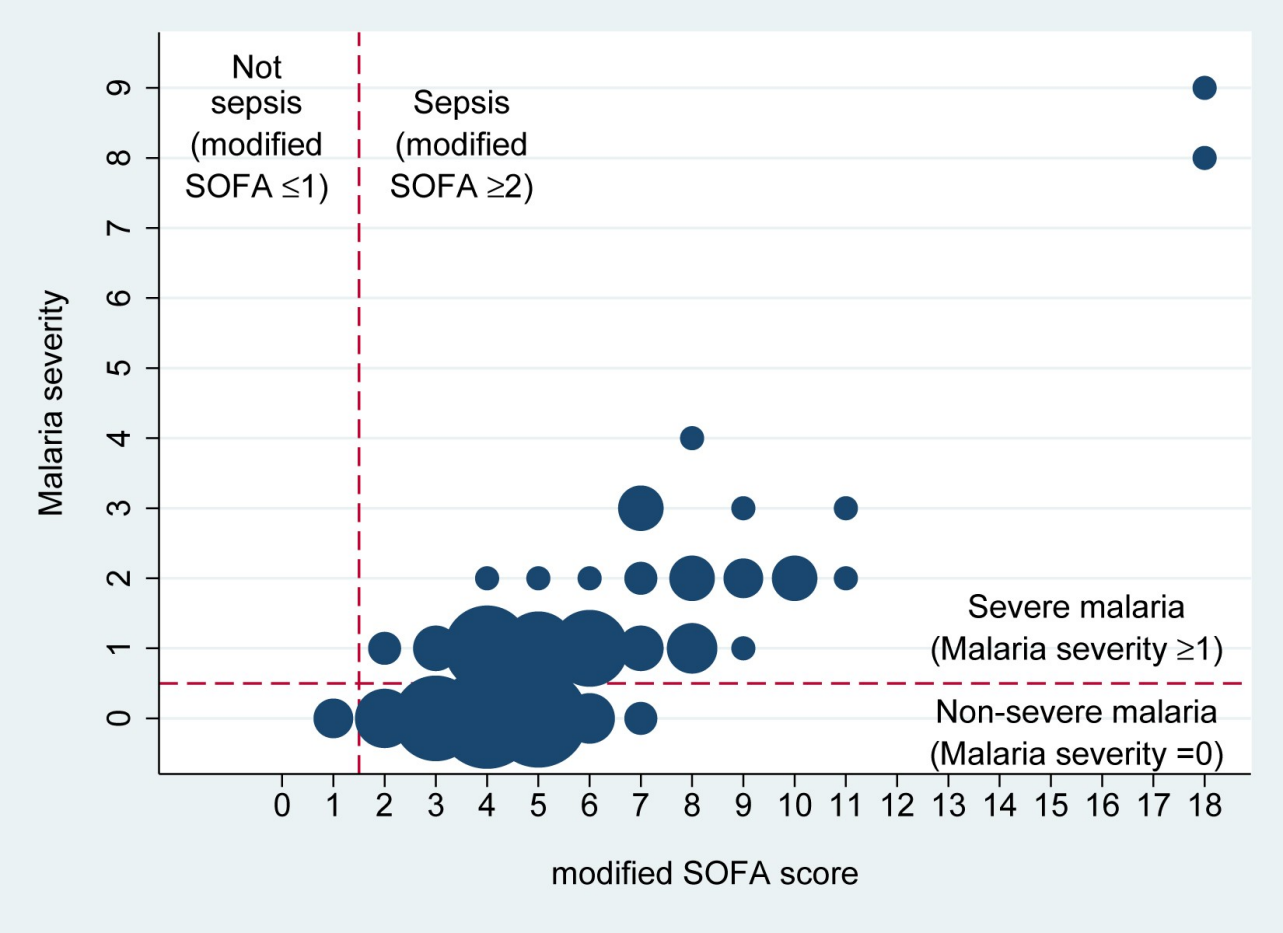
Relationship between modified SOFA score and malaria severity (the total number of WHO severity criteria for severe malaria satisfied by the patient). Size of each dot represents the total number of patients with a modified SOFA score and malaria severity. Each smallest dot represents one patient, and the biggest dot represents 21 patients (modified SOFA score of 4 and malaria severity of 0). Both fatal patients in the study had malaria severity of ≥8.

The modified SOFA score (median 5; IQR 4–6; range 1–18) had a strong correlation with malaria severity (Spearman’s rho = 0.61, p<0.001) (Fig 2). All severe malaria patients were classified as sepsis (100%; 80/80); therefore, the modified SOFA score ≥2 had 100% sensitivity (95%CI 95–100%) to detect severe malaria patients. 96% patients with non-severe malaria in our study (70/73) were classified as sepsis. Among 73 non-severe malaria patients, two (38-year-old male and 57-year-old male) had modified SOFA scores of eight, the highest in the non-severe malaria group. They had systolic blood pressure of 80 mmHg (a modified SOFA score for cardiovascular of 1), creatinine level between 1.2–1.9 mg/dL (a modified SOFA score for renal of 1), bilirubin level between 1.9–2.9 and 1.2–1.9 (a modified SOFA score for liver of 2 and 1, respectively), and had platelets 25,000–50,000/mL or <50,000/mL (a modified SOFA score for coagulation of 3 and 4, respectively). These conditions did not meet the severe malaria definition (Table 1).

### Sensitivity of FilmArray and blood film

A total of 28 malaria-confirmed patients were tested with the FilmArray platform, and 24 of them were positive for the FilmArray. Compared with the nested PCR assay, the diagnostic sensitivity of the FilmArray was estimated to be 86% (24/28; 95%CI, 67% to 96%). The sensitivity was not significantly different by the type of Plasmodium infection (92% [12/13] for PF infection, 77% [10/13] for PV infection and 100% [2/2] for mixed infection; p = 0.70).

Of 152 malaria-confirmed patients who were tested with blood film, as described above, 151 were blood film positive. Compared with the nested PCR assay, the diagnostic sensitivity of blood film was estimated to be 99% (151/152; 95%CI, 96.4–100%).

Of 28 FilmArray-tested cases, 27 were blood film positive and one was blood film negative but FilmArray positive as described above. Therefore, compared with blood film, the diagnostic sensitivity of FilmArray was estimated to be 85% (23/27; 95%CI, 66% to 96%).

## Discussion

Our results show that all severe malaria patients presented as sepsis (defined as a modified SOFA score ≥2), suggesting that the current criteria for sepsis are highly sensitive for the identification of patients who are at high risk of death due to malaria infection. Our results also show that a moderate or higher qSOFA score (qSOFA score ≥1) was sensitive for the detection of severe malaria. Nonetheless, fourteen percent of severe malaria patients presented with qSOFA scores of 1. Based on this lower qSOFA score, these patients may not be considered to have sepsis, and therefore not be afforded the requisite further evaluation–including investigation of the etiology of infection–or management. These findings have important implications for triage and judicious resource allocation in the care of malaria patients. Our findings support the suggestion that any patients presenting with even one of three qSOFA criteria at bedside should prompt careful evaluation for sepsis, initiation of appropriate diagnostic testing and medical interventions, and consideration for referral as appropriate [2]. If the cause of sepsis is malaria, patients should also be evaluated for severe malaria using specific criteria for severe malaria (including WHO criteria for severe malaria [8] and other malaria-specific severity scores [16–20]) and managed using specific guidelines for malaria treatment [8].

The strong correlation between the modified SOFA score and malaria severity is because both criteria are based on five similar parameters as shown in Table 1. However, non-severe malaria patients could be categorized as sepsis because the modified SOFA score includes the coagulation system, which is not present in malaria severity [8], and because the modified SOFA score starts with lower level of bilirubin, blood pressure, Glasgow Coma Scale and creatinine. Thrombocytopenia is not included in the criteria for severe malaria because thrombocytopenia is not an independent predictor of mortality in severe malaria, thrombocytopenia is commonly present in patients with both uncomplicated and complicated malaria, and major bleeding is uncommon in fatal malaria cases [8]. The stricter cut-off for bilirubin, blood pressure, Glasgow Coma Scale and creatinine in malaria comes from large datasets describing the association between those variables and death [8]. In malaria bilirubin is also increased because of intravascular haemolysis, which is intrinsic to the pathophysiology of the disease. The discrepancy is highlighted by the examples of two non-severe malaria patients with modified SOFA scores of 7. Although many criteria for severe malaria are not included in the modified SOFA score (e.g. acidosis, hyperlactatemia, hypoglycemia, etc) [8], we found that none of severe malaria patients were categorized as non-sepsis in our study. This was because any severe malaria patients who had those non-overlapping criteria for severe malaria always had signs of organ dysfunction captured by the modified SOFA score.

Weak to moderate correlation between the qSOFA score and malaria severity is because qSOFA was designed to be simple and available at the bedside without the need for blood tests (to identify hepatic dysfunction, renal dysfunction, acidosis, hyperlactatemia, hypoglycaemia, severe anaemia and hyperparasitaemia) on which the majority of malaria severity criteria are based. Therefore, 14% of severe malaria patients presented with a qSOFA score of 1, and these patients might have not been identified as sepsis or severe malaria patients if a range of blood tests were not performed at the study hospital. Although none of the severe malaria patients presenting with qSOFA score of 1 died in our study, this could be due to the standard of care at the study hospital and the study protocol which evaluated the modified SOFA score on all enrolled patients. The outcome of those patients could be poorer if early detection of sepsis and the identification of pathogenic organisms are delayed. Both fatal cases who met criteria for severe malaria presented with high modified SOFA scores. Parenteral artesunate plus oral mefloquine prescribed in one fatal case is not the treatment regimen recommended for severe malaria by the WHO [8].

Our study also shows that severe PV infection defined by the WHO criteria [8] is not an uncommon cause of malaria in our setting [21]. The sensitivity of FilmArray seems to be high at about 85% in our setting when compared with nested PCR assay or blood film; however, the sensitivity of FilmArray for malaria diagnosis and lower limit of detection still need further evaluation with a larger sample size.

Our study has limitations. First, although we conducted detailed assessments, specific tests were not always available or conducted (e.g. arterial blood gas) and data of doses of adrenergic agents were not recorded. This could lead to an underestimate of the prevalence of sepsis. Second, due to the study design, specificity of qSOFA and modified SOFA scores to detect severe malaria among patients presenting with community-acquired infection was not estimated. This was because most non-severe malaria patients, who should be the main numerator for estimating specificity, were not transferred to the tertiary-care hospital [22] and not included in this study. Third, the modified SOFA score (maximum 23) that we used–because the dosages of dobutamine, dopamine, epinephrine and norepinephrine were not recorded and because arterial blood gases were rarely performed–may be lower than the SOFA score (maximum 24). Nonetheless, the modified SOFA score is strongly associated with mortality of sepsis patients [9]. Fourth, data about blood film and antimalarial treatment prior to and during transportation were not systematically recorded and not presented. Fifth, due to the limited sample size of fatal malaria cases in our study, we could not evaluate whether the qSOFA score, modified SOFA score, WHO criteria for severe malaria [8] and other malaria-specific scores [16–20]) predict fatal outcomes differently or not. However, it is likely that malaria-specific scores could provide higher prognostic indices for malaria fatality and could be selected based on resource availability such as laboratory testing [18].

### Conclusion

This study fills a gap in our knowledge about the presenting manifestations of sepsis among patients with non-severe and severe malaria in LMICs, how qSOFA and SOFA score may be useful in the clinical setting, and how these scores correlate with the criteria of severe malaria defined by the WHO.

## Supporting information

S1 Table Systemic manifestation of infection criteria used for screening. (DOCX)

S1 Fig Study flow diagram. (TIF)
