# Supplementary material for: Utility of qSOFA and modified SOFA in severe malaria presenting as sepsis
Source: PLoS One. 2019 Oct 9;14(10):e0223457. doi: 10.1371/journal.pone.0223457 (PMC6785116; doi:10.1371/journal.pone.0223457)
Supplement: S1 Table — (DOCX) [file pone.0223457.s001.docx]

**Supporting Information**

**S1 Table. Systemic manifestation of infection criteria used for screening**

**General parameters**

1. Fever or hypothermia (Core body temperature defined as > 38.3C or < 36.0C)
2. Tachycardia (heart rate > 90 beats per minute)
3. Tachypnea (respiratory rate > 20 per minute)
4. Altered mental status with Glasgow Coma Score (GCS) < 15 or <10 if intubated
5. Hyperglycemia (plasma glucose > 140 mg/dL) in the absence of diabetes

**Inflammatory parameters**

1. Leukocytosis (white blood cell count > 12,000/µL), leukopenia (white blood cell count < 4000/µL) or immature forms > 10%
2. Plasma C-reactive protein >2 SD above the normal value
3. Plasma procalcitonin >2 SD above the normal value

**Hemodynamic parameters**

1. Arterial hypotension (systolic blood pressure (SBP) < 90 mmHg, mean arterial pressure (MAP) < 70 mmHg, or SBP decrease > 40 mmHg)

**Organ dysfunction parameters**

1. Low oxygen saturation determined by pulse oximetry (SpO2 <95%) determined by pulse oximetry
2. Arterial hypoxemia (PaO2 / FIO2 < 300)
3. Acute oliguria (urine output < 0.5 mL/kg/hr or 45 mmol/L for 2 hours)
4. Creatinine increase > 0.5 mg/dL
5. Coagulation abnormalities (international normalised ratio >1·5 or activated partial thromplastin time >60 seconds)
6. Thrombocytopenia (Platelet count < cells 100,000/µL)
7. Ileus (absent bowel sounds)
8. Hyperbilirubinaemia (plasma total bilirubin > 4 mg/dL)

**Tissue perfusion parameters**

1. Hyperlactatemia (> 1 mmol/L)
2. Decreased capillary refill or mottling
3. Significant edema or positive fluid balance
