# Supplementary figures and images for: Utility of qSOFA and modified SOFA in severe malaria presenting as sepsis
Source: PLoS One. 2019 Oct 9;14(10):e0223457. doi: 10.1371/journal.pone.0223457 (PMC6785116; doi:10.1371/journal.pone.0223457)

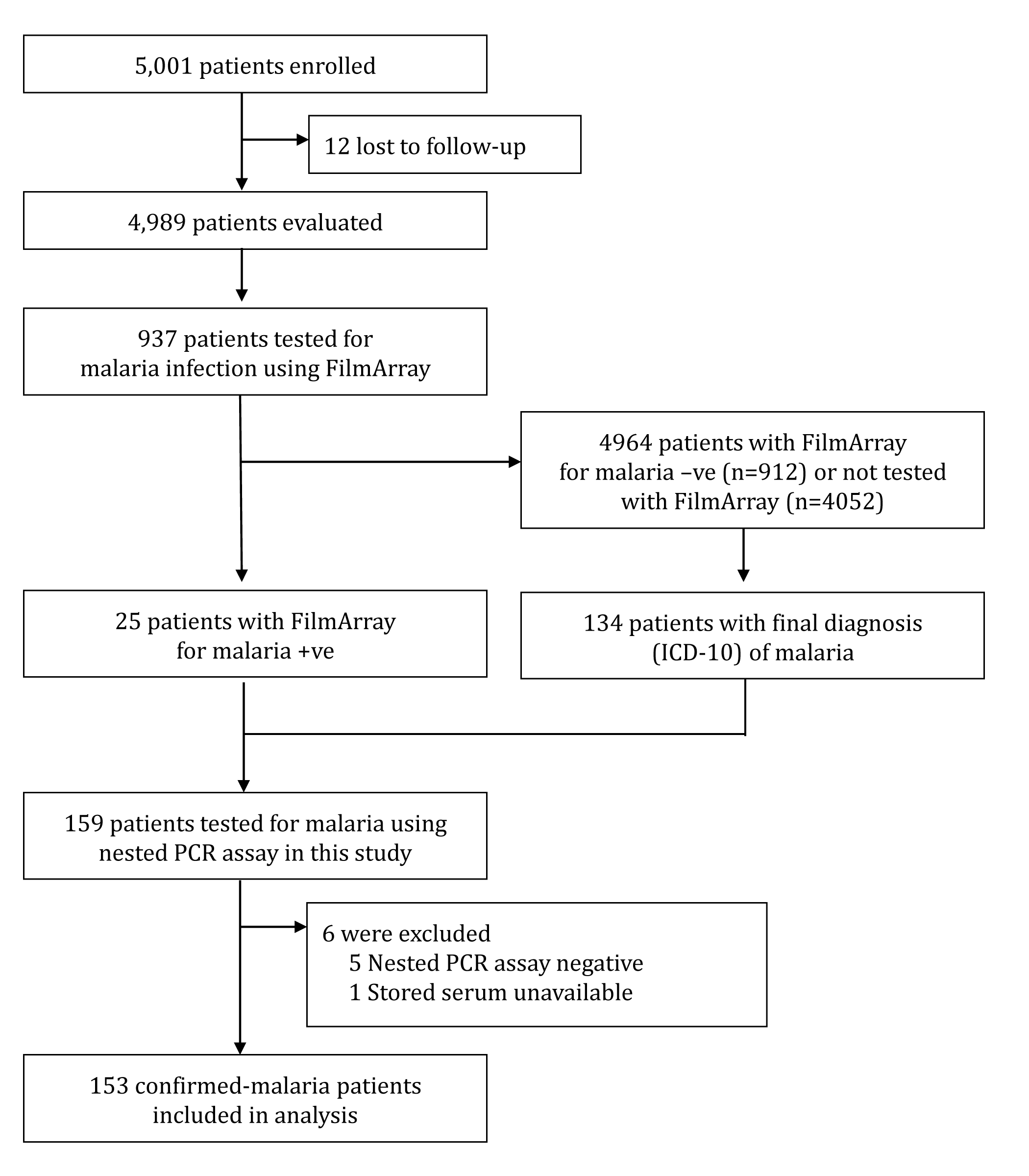

Supplement: S1 Fig — (TIF) [file pone.0223457.s002.tif]
